# Structural Basis for the Regulation Mechanism of the Tyrosine Kinase CapB from Staphylococcus aureus

**DOI:** 10.1371/journal.pbio.0060143

**Published:** 2008-06-10

**Authors:** Vanesa Olivares-Illana, Philippe Meyer, Emmanuelle Bechet, Virginie Gueguen-Chaignon, Didier Soulat, Sylvie Lazereg-Riquier, Ivan Mijakovic, Josef Deutscher, Alain J Cozzone, Olivier Laprévote, Solange Morera, Christophe Grangeasse, Sylvie Nessler

**Affiliations:** 1 Laboratoire d'Enzymologie et Biochimie Structurales, CNRS, Gif sur Yvette, France; 2 Institut de Biologie et Chimie des Protéines, CNRS, Université Lyon 1, Université de Lyon, Lyon, France; 3 Institut de Chimie des Substances Naturelles, CNRS, Gif sur Yvette, France; 4 Center for Microbial Biotechnology, BioCentrum, Technical University of Denmark, Lyngby, Denmark; 5 Laboratory of Microbiology and Molecular Genetics, AgroParisTech, CNRS, INRA, Thiverval-Grignon, France; Harvard University, United States of America

## Abstract

Bacteria were thought to be devoid of tyrosine-phosphorylating enzymes. However, several tyrosine kinases without similarity to their eukaryotic counterparts have recently been identified in bacteria. They are involved in many physiological processes, but their accurate functions remain poorly understood due to slow progress in their structural characterization. They have been best characterized as copolymerases involved in the synthesis and export of extracellular polysaccharides. These compounds play critical roles in the virulence of pathogenic bacteria, and bacterial tyrosine kinases can thus be considered as potential therapeutic targets. Here, we present the crystal structures of the phosphorylated and unphosphorylated states of the tyrosine kinase CapB from the human pathogen Staphylococcus aureus together with the activator domain of its cognate transmembrane modulator CapA. This first high-resolution structure of a bacterial tyrosine kinase reveals a 230-kDa ring-shaped octamer that dissociates upon intermolecular autophosphorylation. These observations provide a molecular basis for the regulation mechanism of the bacterial tyrosine kinases and give insights into their copolymerase function.

## Author Summary

An idiosyncratic new class of bacterial enzymes, bacterial tyrosine-kinases (BY-kinases), has been characterized. These enzymes, which are involved in an increasing number of physiological processes ranging from stress resistance to pathogenicity, share no sequence similarities with eukaryotic kinases, and their function remains largely unknown. They have nevertheless been described to undergo autophosphorylation on a C-terminal tyrosine cluster and to phosphorylate endogenous protein substrates. We describe here the first crystal structure of a bacterial tyrosine kinase, namely CapB from the pathogen *Staphylococcus aureus*, in complex with the cytoplasmic domain of the transmembrane stimulatory protein CapA. Our data explain the activation mechanism of CapB by CapA and allow us to propose a regulatory mechanism based on intermolecular autophosphorylation. These results also give new insights onto the phosphorylation of the endogenous substrate CapO, an enzyme involved in the synthesis of polysaccharide precursors. CapA and CapB, among others, are involved as copolymerases in the synthesis of extracellular polysaccharides that are thought to be potent virulence factors. Thus, these structural data provide the basis for designing specific inhibitors for these enzymes, which constitute an original and attractive target for the development of new drugs to treat infectious diseases.

## Abstract 3

Structural analysis of a conserved bacterial tyrosine kinase from*Staphylococcus aureus* provides the basis for deciphering its regulatory mechanism, leading to a model for its implication in extracellular polysaccharide synthesis.

## Introduction

Protein phosphorylation–dephosphorylation represents one of the most powerful and versatile mechanisms of molecular regulation in living organisms. For many years, however, protein phosphorylation–dephosphorylation was considered to be exclusive to eukaryotes. It was only after a long period of controversy that the existence of this modification was documented in bacteria (for a review, see [1]). Early studies essentially focused on the characterization of the “two-component system” [2] and “phosphotransferase PTS system” [3], the well-known hallmarks of bacterial signalling and regulation in which proteins are phosphorylated on histidines and aspartic acids. Then, thanks in large part to genomics, the widespread presence of genes encoding eukaryotic-like serine/threonine kinases [4] and phosphatases has also turned out to be indisputable in bacteria [5]. In addition, high-accuracy mass spectrometry experiments have recently allowed the characterization of more than 100 serine, threonine, and tyrosine phosphorylation sites in two model bacteria [6,7].

Besides, some bacterial members of the large family of P-loop containing proteins [8,9] characterized by the Walker A nucleotide binding motif [10] were found to carry a protein kinase activity [11]. The first structurally [12] and functionally [13] characterized member of this new family of P-loop containing protein kinases was a serine kinase, the bifunctional HPr kinase/phosphorylase involved in a signalling pathway regulating the use of carbon sources by bacteria [14].

Progress on tyrosine phosphorylation in bacteria was slower, and it was only in 1997 that the first gene encoding a bacterial tyrosine kinase was characterized [15]. This enzyme is structurally and functionally unrelated to its eukaryotic counterparts. Like the HPr kinase/phosphorylase, it belongs to the family of P-loop containing protein kinases. This particular type of tyrosine kinase has been identified in numerous bacteria [16], thus defining a bacterial idiosyncratic family of bacterial tyrosine kinases (BY-kinases) [17].

**Figure 1:**
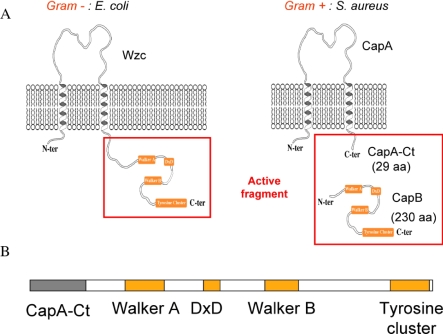
Active Fragment of BY-Kinases (A) Schematic organisation of bacterial tyrosine kinases. The Walker A and B motifs, as well as the catalytic DxD motif and the tyrosine cluster, are represented as orange boxes. The cytoplasmic active fragments are indicated by red frames. aa, amino acids. (B) Construction of the active CapAB chimeric protein linking the C-terminal cytosolic fragment 194a–222a of CapA1 to CapB2 (residues 1b–230b).

In proteobacteria and actinobacteria, BY-kinases are encoded as a single polypeptide, whereas in Firmicutes, they are found in the form of two interacting proteins. The periplasmic N-terminal and the cytoplasmic C-terminal domains of BY-kinases from proteobacteria and actinobacteria are homologous to the membrane adaptor and the cytoplasmic BY-kinase from Firmicutes, respectively (Figure 1A). All BY-kinases that have been examined undergo autophosphorylation on a C-terminal tyrosine cluster, but also phosphorylate other proteins. Among the first identified endogenous protein substrates of BY-kinases were proteins involved in polysaccharide production [18–20], but also RNA polymerase sigma factors [21] and single-stranded DNA binding proteins [22]. Therefore, BY-kinases are implicated in many other important physiological processes, including stress response, DNA metabolism, antibiotic resistance, control of the bacterial cell cycle, and pathogenicity [17,23].

The mechanism by which BY-kinases control extracellular polysaccharide biosynthesis is the best documented. Since 2000, an increasing number of publications have analyzed this process, and tyrosine phosphorylation has turned out to be a key feature of capsule formation [24]. BY-kinases have been characterized as polysaccharide copolymerases (PCP) belonging to multiprotein transmembrane machineries involved in synthesis and/or export of a large number of extracellular polysaccharides [25]. However, the accurate function of their phosphorylation remains unclear even though it has been shown to influence both the length and the amount of the produced polymer [26–28], thus modifying the physicochemical properties of the capsule [29]. In bacterial human pathogens such as Staphylococcus aureus, capsule promotes virulence in animal models of infection [30,31]. The S. aureus capsule has been shown to be involved in protection against phagocytosis [32] and in modulation of the host immune response [33]. Therefore, structural analysis of this new type of enzyme would not only contribute to depict their function in extracellular polysaccharide synthesis, but could also be used as a basis for a structure-based drug design project targeting microbial pathogens.

In this work, we focused on S. aureus serotype 5, a Gram-positive pathogen responsible for a diverse spectrum of animal and human diseases, and predominating in clinical isolates [31,34]. S. aureus whole-genome analysis [35] revealed two couples of cytoplasmic BY-kinase and associated transmembrane adaptor: Cap5A1/Cap5B1, encoded by genes located in the *cap* operon controlling capsule biosynthesis, and the highly similar Cap5A2/Cap5B2 couple, the genes of which are located elsewhere on the genome [36]. Surprisingly, it has been demonstrated that the Cap5B2 tyrosine kinase activity is more efficiently activated by the transmembrane protein Cap5A1 than by the cognate Cap5A2 activator [36]. More precisely, the last 29 C-terminal and cytoplasmic residues of Cap5A1 (or Cap5A2) are sufficient for stimulation of the kinase activity. Once activated, Cap5B2 trans-phosphorylates on its tyrosine cluster, but also phosphorylates Cap5O, an UDP-acetyl-mannosamine dehydrogenase involved in the production of a polysaccharidic capsule precursor [20]. Despite the 57% overall sequence identity with Cap5B2 and the high conservation of the tyrosine cluster, no kinase activity could be detected for Cap5B1, either in the presence of Cap5A1 or of Cap5A2 [36].

Reproducing the Gram-negative organization of BY-kinases by direct linkage of the Cap5A1Ct fragment (herein called CapACt) to the N-terminal extremity of Cap5B2 (herein called CapB) allowed the production of a fully active soluble protein called CapAB (Figure 1B). We performed a high-resolution structural analysis of this chimeric protein and of its inactive P-loop mutant CapAB(K55M). The respective 1.8 Å and 2.6 Å resolution structures provide the first atomic view of a tyrosine kinase from bacterial origin. The regulatory autophosphorylation mechanism suggested by this structural analysis was further investigated by mutational and biochemical approaches. We finally propose a molecular model for the regulation of extracellular polysaccharide synthesis.

## Results

### First Crystal Structure of a Bacterial Tyrosine Kinase

The structure of the S. aureus CapAB chimeric protein was determined at 1.8 Å resolution (Table 1). Evolutionary classification of the P-loop proteins suggested that the BY-kinase family is closely related to the Mrp-MinD subfamily P-loop ATPases [37]. A CapA/CapB mutational analysis based on the structure of the MinD cell division regulator confirmed this similarity [36]. Thus, despite the low sequence identity (17%) between S. aureus Cap5B2 and the Pyrococcus horikoshii MinD protein [38], the later was successfully used as the starting model in the molecular replacement procedure that allowed us to solve the first BY-kinase structure.

**Table 1:**
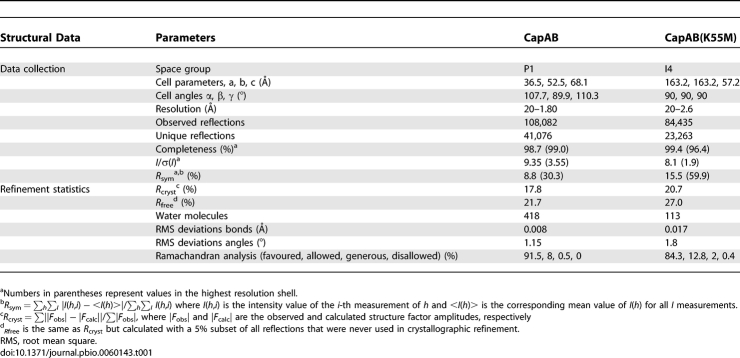
Structural Data

The refined structure of the CapAB chimeric protein includes a continuous polypeptide corresponding to residues 197a to 222a of Cap5A1 linked to residues 1b to 215b of Cap5B2. The N-terminal extremity (6His-tag and Cap5A1 residues 194a–196a) and the C-terminal tyrosine cluster (Cap5B2 residues 215b–230b) are disordered. The asymmetric unit contains two molecules with a small contact surface area of about 600 Å^2^, characteristic of packing interactions [39]. The crystal form of the truncated cytoplasmic domain of CapAB is thus a monomer, as observed in solution by size-exclusion chromatography (unpublished data).

**Figure 2:**
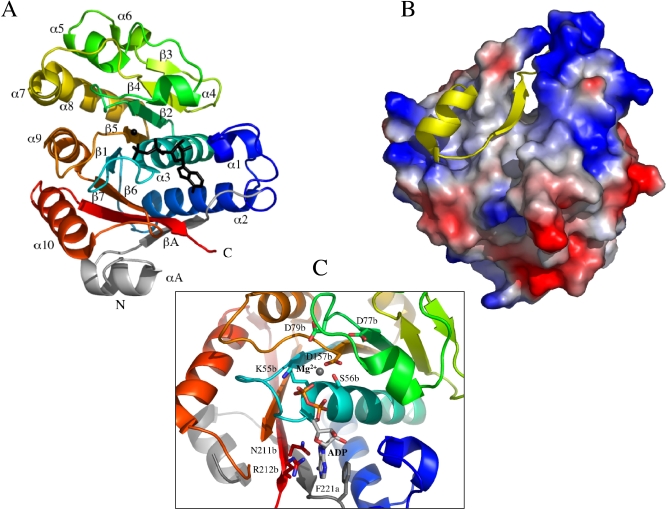
Structure of the Phosphorylated CapAB Monomer (A) Overall structure of the CapAB monomer. At the N-terminus of the chimeric molecule, CapACt is shown in grey, linked to CapB coloured in a rainbow spectrum from blue to red toward the C-terminal extremity. The ADP molecule is shown in black sticks, and the associated magnesium ion as a black sphere. The P-loop is in cyan. The secondary structure elements are labelled. (B) CapB-CapACt interaction. A yellow cartoon trace of CapACt is shown on the surface of CapB coloured accordingly to electrostatic potential. (C) Close view of the active site. The ADP molecule and the catalytic residues K55b, S56b, D77b, D79b, and D157b are shown in sticks coloured by atom type, as well as residues N211b, R212b, and the CapACt residue F221a interacting with the base moiety of the nucleotide.

The CapB kinase core possesses a P-loop type α/β mononucleotide binding fold (Figure 2A). A central seven-stranded β-sheet with the strand order 3425167 is flanked on one side by four (α1–α4) and on the other by six (α5–α10) α-helices. The distal strand β3 (residues 111b–113b) of the β-sheet is antiparallel to the others. The loop connecting strand β1 to helix α3 is the phosphate-binding loop (P-loop) corresponding to a divergent Walker A motif (E_49_APGAGKS_56_).

The CapACt fragment (residues 194a–222a) provides an additional strand called βA (residues 214a–219a) interacting with strand β7 of CapB and thus completing the central β-sheet of the protein. CapACt also contains an additional α-helix called αA (residues 202a–211a) interacting with helix α10 (residues 187b–200b) of CapB. The total surface contact area between CapACt and CapB covers about 3,000 Å^2^ and is highly hydrophobic (Figure 2B), explaining why CapB is poorly soluble in the absence of CapACt (unpublished data).

Structural comparison with the Protein Data Bank using the SSM service at the European Bioinformatics Institute [40] confirmed that the closest structural relative of the chimerical CapAB is the bacterial cell division regulator MinD. A *Z*-score of 9.2 with a root mean square deviation of 1.94 Å over 160 aligned Cα atoms was obtained with the P. horikoshii MinD structure [38] used in the molecular replacement procedure. Comparison of the two structures is presented in Figure S1. The active sites of both proteins are highly similar, and the nucleotide-binding mode is conserved. In particular, the base is in sandwich interactions between the conserved arginine and a hydrophobic residue from MinD helix α9 replacing CapACt F221a. However, whereas MinD displays an ATPase activity [41], we verified that the protein does not autophosphorylate and is unable to phosphorylate UgD, the substrate of the Escherichia coli BY-kinase, Wzc (unpublished data).

### CapACt Participates in Nucleotide Binding

The active site of the protein contains ADP-Mg, most probably resulting from the hydrolysis of the 10 mM ATP-Mg contained in the crystallization solution. Typical interactions are observed between the phosphate groups of ADP and the P-loop. The associated magnesium ion is fully chelated by the side chain of S56b, the β-phosphate, and four water molecules. The latter are stabilized by an interaction network involving three conserved aspartate residues, i.e., D157b from the Walker B motif and D77b, D79b from the conserved DxD motif located 20 residues downstream from the Walker A motif. The O4 oxygen atom of the ribose interacts with CapB residue R212b, whereas the N6 and N7 nitrogen atoms of the adenine ring specifically interact with the side chain carboxyamide of residue N211b. The adenine ring is also involved in a classical hydrophobic sandwich interaction with the side chains of CapB R212b and CapA F221a (Figure 2C).

The stimulatory effect of CapACt on the kinase activity of CapB has been shown to be correlated with an increased affinity for ATP [36]. The (F221A) mutation was introduced in the CapACt segment of the chimeric CapAB protein in order to verify the essential role of residue F221a suggested by the structure. A strongly decreased autokinase activity was observed with the CapAB(F221A) mutant compared to the wild-type CapAB protein (Figure S2). Preliminary fluorescence experiments using labelled nucleotide analogs further confirmed that this loss of activity is correlated with a reduced affinity of the CapAB(F221A) mutant for the nucleotide (unpublished data).

### The Monomeric Form of CapAB Is Phosphorylated

**Figure 3:**
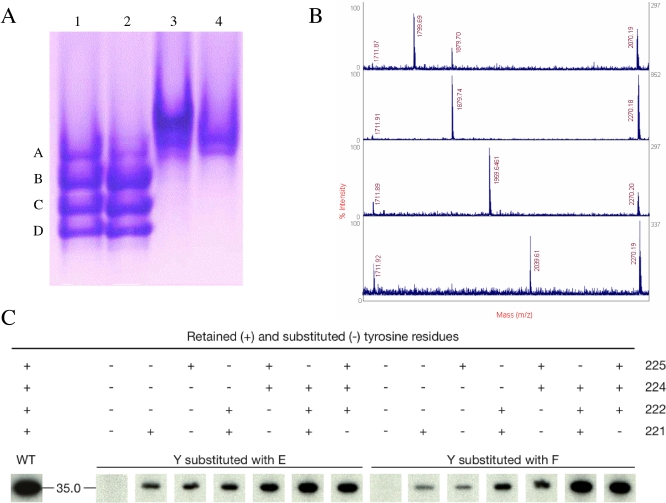
Phosphorylation State Analysis (A) Blue native PAGE analysis of CapAB. Lane 1, purified CapAB protein used for crystallization; Lane 2, CapAB incubated with ATP-Mg; Lane 3, CapAB incubated with the S. aureus phosphatase CapC2; and Lane 4, nonphosphorylated inactive CapAB(K55M) mutant protein. (B) MALDI-TOF mass spectrometry phosphopeptide identification. Respective MS spectra of the four bands (A, B, C, and D) from lane 1 of the native gel after nanoscale Fe(III)-IMAC purification of the phosphorylated tryptic peptide (CapB residues D216b–E228b). The peaks corresponding to phosphorylated ions are highlighted by a red box. Bands A, B, C, and D, respectively, contain the mono-, di-, three-, and four-phosphorylated ions characterized by *m/z* values of 1,799.7*m/z*, 1,879.7*m/z,* 1,959.6*m/z*, and 2,039.6*m/z*. A small contamination of band A by band B is observed. Other labelled peaks correspond to nonphosphorylated tryptic peptides, these *m/z* peaks represented “false positives” retained by IMAC column because of the presence of acidic residues or Ser in their peptide sequence. The peak = 1733.8 is a nonspecific tryptic peptide. (C) Autoradiogram of in vitro phosphorylated tyrosine-mutated forms of CapAB separated by SDS-PAGE electrophoresis. The tyrosine residues retained (+) or substituted (−) either in F (to mimic an unphosphorylated state) or E (to mimic a phosphorylated state), are indicated above each lane. The wild-type form is indicated by WT.

The absence of electron density for the CapB tyrosine cluster raised the question of its phosphorylation state. Analysis of the CapAB protein sample from the crystallisation experiments using nondenaturing gel electrophoresis revealed four bands of about equivalent intensities (Figure 3A). These four bands were converted in a single, slowly migrating band when the CapAB sample was treated with the cognate S. aureus tyrosine phosphatase CapC2. The inactive CapAB(K55M) mutant, affected at the catalytic K55b residue from the conserved Walker A motif [36], also displays a single band (Figure 3A). These results strongly suggest that the crystallized CapAB sample is heterogeneously phosphorylated.

This hypothesis was further investigated by analyzing each band of the gel using mass spectrometry. This analysis confirmed that the upper band observed with the inactive mutant or after dephosphorylation of CapAB by CapC indeed corresponds to the unphosphorylated protein. The four other bands correspond to one, two, three, and four phosphorylations from top to bottom, respectively (Figure 3B). Tandem mass spectrometry analysis and sequencing of each phosphopeptide allowed us to calculate the percentage of phosphorylation for each tyrosine (unpublished data). Any combination of phosphorylated tyrosines was observed in each band. For example, the distribution of population for the monophosphorylated peptide was evaluated as 12% Y225, 28% Y224, 19% Y222, 35% Y221, and 6% nonphosphorylated.

In parallel, 14 proteins containing various combinations of Tyr to Phe or Tyr to Glu exchanges (Glu was expected to mimic a phosphorylation) have been engineered to further assess the influence of each tyrosine on the autokinase activity of CapAB. This mutational analysis revealed that each of the four tyrosines is phosphorylated independently of the F or E mutation applied to the three other tyrosines (Figure 3C). These results confirm the heterogeneous autophosphorylation process of CapAB, and indicate that phosphorylation occurs without any preferred order.

Thus, the crystallized protein was heterogeneously phosphorylated, and the absence of electron density corresponding to the C-terminal extremity suggests that the phosphorylated tyrosine cluster is highly flexible.

### Unphosphorylated CapAB(K55M) Forms a Ring-Shaped Octamer

**Figure 4:**
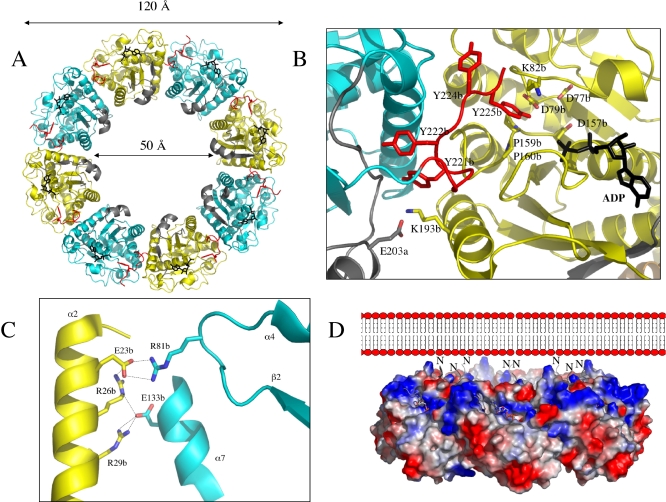
Structure of the Unphosphorylated CapAB(K55M) Octamer (A) Octameric ring-shaped structure. The CapB(K55M) subunits are shown as cartoon traces alternately coloured in cyan and yellow with the tyrosine cluster highlighted in red. The side chain of Y225b pointing in the active site of the neighbouring subunit is shown in red sticks. The fused CapACt fragments are shown in grey, and the bound nucleotides in black sticks. The inner and outer diameters of the ring are given. (B) Subunit interactions. The view is centred at the active site of a yellow subunit with the bound ADP-Mg molecule and the catalytic residues highlighted in sticks. The neighbouring interacting molecule is in cyan with the C-terminal cluster in red. The four tyrosines are shown in sticks with Y225b pointing into the active site. (C) Close view of the conserved specific contacts. The conserved H-bonds between helix α2 residues E23b, R26b, and R29b of a yellow subunit and residues E133b and R81b from a cyan subunit are shown as dashed lines. (D) Orientation of the octamer. In this side view of the ring, represented with electrostatic potential surfaces, the N-terminal extremity (labelled N) of the chimeric protein corresponding to the C-terminal juxtamembrane part of CapA are located on a positively charged surface (in blue) of the octamer facing the negatively charged (in red) phospholipid layer of the membrane. The active sites highlighted by the nucleotide shown in sticks are exposed on the external surface of the ring.

The structure of the inactive P-loop CapAB(K55M) mutant was determined to 2.6Å resolution (Table 1). It presents a subunit fold highly similar to that of the CapAB wild-type protein with an rmsd of 0.67 Å over 241 aligned residues. The major difference concerns the quaternary structure of this unphosphorylated form of the protein that associates to a ring-shaped octamer (Figures 4A). The total surface contact area of about 2,000 Å between two neighbouring subunits is characteristic of biological interactions [39].

Residues 216b–228b of the tyrosine cluster that were disordered in the phosphorylated wild-type structure form a long loop with Y225b side chain fitting in the active site of the neighbouring CapB molecule (Figure 4B). Despite the absence of a nucleotide in the crystallization solution, ADP-Mg is observed in the P-loop of the CapAB(K55M) mutant protein. This is in agreement with a previous study showing that in P-loop proteins, this mutation inhibits the phosphate transfer but increases the affinity of the protein for the nucleotide [42]. The bound ADP molecule thus originates from the E. coli cell extract and remains bound to the protein during the purification process.

The C-terminal cluster and helix α2 of one subunit form 44% and 50%, respectively, of the contact area facing the surface loops β2-α4, β4-α7, and β5-α9, as well as helix α10 and the P-loop of the neighbour subunit. With the exception of a specific interaction between the αA residue E203a and the helix α10 residue K193b from an adjacent subunit, CapACt is not directly involved in octamer contacts (Figure 4B).

### Autophosphorylation Mechanism

The side chain of Y225b is bound in the active site of the neighbour subunit via hydrophobic sandwich interactions with the side chain of K82b and the two consecutive proline residues P159b–P160b forming an extended hhhhDTPP Walker B motif characteristic of the BY-kinase subfamily of P-loop ATPases (Figure S3). The hydroxyl group of Y225b is pointing toward the 4.8 Å distant β-phosphate of the bound ADP molecule (Figure 4B). Superimposition with the Pyrococcus furiosus MinD/AMP-PCP complex [41] showed that this Y225b hydroxyl group is 2.84 Å from the γ-phosphate to be transferred (unpublished data). The catalytic residue D79b from the conserved DxD motif is positioned to deprotonate the Y225b hydroxyl, a prerequisite for its phosphorylation (Figure 4B).

## Discussion

### Specificity of the Autophosphorylation Process

The intermolecular autophosphorylation process suggested by the CapAB(K55bM) octameric structure is in agreement with all BY-kinases phosphorylation data published so far, even those originally thought to reflect an intramolecular mechanism [19]. However, the exact autophosphorylation process remains unclear. Although the structure suggests that Y225b is preferentially phosphorylated, our mass spectrometry and site-directed mutagenesis experiments revealed a heterogeneous phosphorylation pattern of the four tyrosines without any preferred order.

The tyrosine cluster forms a long surface loop with a putative high intrinsic flexibility, suggesting that it could adopt different conformations. After intermolecular autophosphorylation, the cluster most probably exits the neighbouring active site, inducing dissociation of the kinase domains, as observed in the monomeric structure of the phosphorylated CapAB protein. The heterogeneous phosphorylation pattern however suggests that the affinity between subunits with partially phosphorylated clusters is sufficient to allow reassociation and further phosphorylation of the cluster.

Heterogeneous phosphorylation has also been observed in vivo with Wzc of E. coli [43] and CpsD of S. pneumoniae [44]. Whereas the core of the kinase domain is highly conserved among BY-kinases, the sequence and the length of the tyrosine cluster, as well as the number and the relative positions of the phosphorylatable tyrosines, are variable (Figure S3). This variability further supports the hypothesis of a biological nonspecific phosphorylation pattern.

### Biological Relevance of the Octamer

BY-kinases sequence comparison (Figure S3) demonstrated that the CapB helix α2 residues E23b, R26b, and R29b, as well as residues E133 of helixα7 and R81b of loop β2-α4, specifically involved in the octamer contacts are highly conserved among the whole family, except in CpsD from Streptococcus pneumoniae. The conservation of this interface (Figure 4C) suggests that the octamer is functionally important. However, simultaneous replacement of the four conserved interface residues of CapAB helix α2 with alanines did not significantly reduce the autokinase activity of the protein (unpublished data), suggesting that these four residues are not essential for the intermolecular phosphorylation process.

As confirmed by size-exclusion chromatography analysis of the CapAB(K55M) mutant (unpublished data), only the monomeric form of the protein is observed in solution. This result suggests that the octamer is not stable in the absence of the transmembrane domain, except in the high protein concentrations used in crystallization. In solution, autophosphorylation probably occurs via nonspecific transient interactions between subunits. Thus, in our opinion, in vitro experiments performed with truncated soluble proteins poorly represent the in vivo 2-D situation occurring at the membrane surface, where the cytoplasmic kinase domains are maintained close together via specific interactions with the octameric transmembrane activator (Figure 4D).

### Comparison with the Electron Microscopy Structure of E. coli Wzc

Whereas the kinase and transmembrane activator form two distinct proteins in Firmicutes like *S. aureus,* they are associated in a single protein in proteobacteria as in the E. coli Wzc protein (Figure 1). We can thus speculate that in vivo, the octameric ring extends to the transmembrane regulatory domain. This hypothesis is supported by the electron microscopy (EM) low-resolution structures of Wzc solved in the absence [45] and presence [46] of the associated translocon Wza of the extracellular polysaccharide synthesis machinery of E. coli [47]. In both cases, Wzc displays a transmembrane ring-shaped structure with dimensions (≈50-Å inner diameter, ≈120-Å outer diameter) similar to those observed in CapAB (Figure 4A). The cytoplasmic Wzc kinase domains formed four spikes extruding from the transmembrane ring. The Wzc samples used in this EM experiments were phosphorylated. This EM structure is thus in agreement with our results, suggesting that only the nonphosphorylated cytoplasmic kinase domains associate into a ring-shaped octamer favouring intermolecular autophosphorylation. The tetrametric symmetry used in the EM analysis extends to the ring-shaped Wza component of the Wzc-Wza complex. However, a high-resolution crystal structure of Wza clearly demonstrated that the translocon is an octamer [48]. It is thus possible that Wzc, like CapAB, also forms an octameric ring allowing intermolecular autophosphorylation of the subunits before dissociation of the cytoplasmic kinase domains. The association–dissociation process of the cytoplasmic region of the ring is most probably cooperative.

### Insights into the Molecular Mechanism Regulating Capsule Production

Both the phosphorylated and dephosphorylated forms of Wzc have been shown to influence the synthesis of polysaccharides [16]. However, a positive regulation of the polysaccharide synthesis by PCPs phosphorylation has been observed in certain bacterial strains [49,50], whereas the opposite situation has been found in other strains in which the nonphosphorylated form of PCPs allows polysaccharide production [27,28,44]. These contradictory data support the hypothesis that polysaccharide synthesis requires both the phosphorylated and unphosphorylated forms of PCPs. Bacterial-encoded phosphotyrosine phosphatases catalyze the dephosphorylation of PCPs [51,52]. Thus, cycling between both forms of PCPs represents an attractive model [28,53].

**Figure 5:**
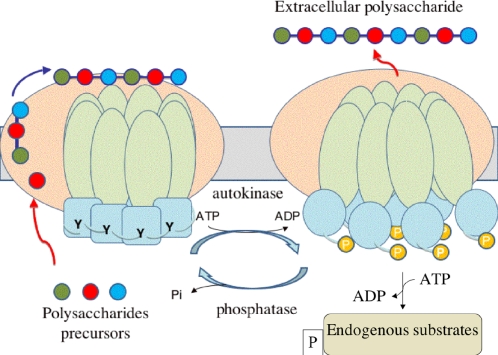
Model of the Copolymerase Cycling Activity The PCP is represented as an octamer with the transmembrane domain in green and the kinase domain in blue. In the close conformation on the left side of the figure, the unphosphorylated kinase domains associate in an octameric ring, with each tyrosine tail (Y) interacting with the active site of the neighbouring subunit. This ring-shaped structure extends to the transmembrane domain, interacting with other components of the polysaccharide assembly machinery represented in salmon. The polysaccharide precursors are shown as coloured balls polymerized and exported via the machinery. In the open conformation on the right side of the figure, the phosphorylated kinase domains (P) resulting from the ATP-dependent autokinase activity are dissociated, thus releasing the constraints on the transmembrane domains. This conformation switch induces changes in the interactions with the other component of the assembly machinery and/or in the affinity for the polysaccharides. The activated kinase domains further phosphorylate endogenous substrates represented in beige. The PCP is then dephosphorylated by its cognate phosphatase following a cyclic process supporting the polymerisation and export mechanism of polysaccharides.

Our data further suggest a structural-mechanical cycling process according to which phosphorylation/dephosphorylation of PCPs would induce cyclic dissociation–association of the cytoplasmic ring-shaped octamer (Figure 5). This conformational switch would most probably be transmitted to the transmembrane domain of PCPs that would be affected in its interaction with the other protein components of the polysaccharide assembly complex, such as the polysaccharide unit polymerase, the lipid-linked repeat unit flippase, or the lipid-sugar transferase [24]. However, one cannot exclude that the affinity of the machinery for the nascent polysaccharide [54] would also be affected. Cycling phosphorylation/dephosphorylation of PCPs would thus adjust continuously the polymerization and export steps of the polysaccharidic polymer. Given the strong conservation between PCPs, this may be a general model for the regulation of extracellular polysaccharide synthesis.

Our model suggests that the juxtamembrane fragments preceding αA-βA in the CapA structure are acting as flexible linkers allowing the phosphorylated kinase domains to dissociate from each other while remaining associated with the octameric transmembrane modulator. This phosphorylated open conformation would be the active form for phosphorylation of the endogenous protein substrate. This hypothesis is supported by CapO phosphorylation experiments using the Glu substituted form of the CapAB tyrosine cluster mimicking the phosphorylated state of the protein. On the other hand, a deleted form of the protein missing the terminal tyrosine cluster is still able to phosphorylate CapO, thus demonstrating that the phosphorylated tyrosine cluster is not directly implicated in the interaction with CapO (Figure S4).

### Perspectives

The involvement of the BY kinases, not only in capsule production, but also in other important cellular processes implicated in the virulence of bacterial pathogens [17], designates them as potential therapeutic targets. Controlling the development and the adaptation ability of bacteria, and more specifically of pathogens, is a major scientific challenge. Blocking these bacterial idiosyncratic tyrosine kinases represents, therefore, an original and attractive strategy with expected limited side effects on the host cells and potentially important biomedical applications.

## Materials and Methods

### Mutagenesis, protein expression, and purification.

The previously described plasmids pQE30-A1CtB2 and pQE30-A1CtB2K [36] were used to produce the His-tagged chimeric wild-type protein CapAB and the inactive mutant CapAB(K55M), respectively. Wild-type or mutated chimeric CapAB proteins contain the last 29 C-terminal and cytoplasmic residues 194a–222a of Cap5A1 His-tagged at the N-terminus, whereas the C-terminus was fused to full-length wild-type or mutant Cap5B2 (residues 1b–230b). Site-directed mutagenesis of CapAB was carried out by PCR amplification using specific primers (Table S1). The same strategy was applied to create CapAB mutants deleted of the C-terminal tyrosine cluster (residues Y221b–S230b). The plasmid pQE30-CapO described in [20] was used to produce the S. aureus protein substrate CapO. The S. aureus phosphosphotyrosine phosphatase CapC2 was PCR-amplified using specific primers (Table S1) and inserted into vector pET15b. Proteins were overproduced in E. coli, purified by IMAC and size-exclusion chromatography (Superdex S75), concentrated by centrifugal ultrafiltration, and stored at −20 °C.

### Nondenaturing electrophoresis.

Prior loading on a nondenaturing 12% polyacrylamide gel, 45 μM CapAB samples were respectively incubated for 3 h at 37 °C alone, with 1 mM ATP/MgCl_2_ or with 8 μM recombinant His-tagged S. aureus CapC2 phosphatase and 1 mM MnCl_2_ [55]. After gel migration, the proteins were stained with Coomassie Blue.

### Matrix-assisted laser desorption/ionization time-of-flight mass spectrometry analysis.

Stained protein bands from the native gel were excised and washed with 25 mM NH_4_CO_3_. In-gel tryptic digestion was performed following the classical protocol [56]. The phosphopeptides were purified by nanoscale Fe(III)–Immobilized Metal Ion Affinity Chromatography (IMAC) according to the manufacturer's (Millipore) instructions. The tryptic peptides were loaded onto the column, and after extensive washing, phosphopeptides were eluted in 5 μl of 2% NH_4_OH. Mass spectra were recorded in positive reflectron mode with a matrix-assisted laser desorption/ionization time-of-flight MALDI-TOF/TOF 4800 mass spectrometer (Applied Biosystem) using a-cyano-4-hydroxycinnamic acid (Sigma) as a matrix. Close calibration was performed using angiotensin I ([M+H^+^], 1296.68) and adrenocorticotropic hormone 18–39 (M+H^+^, 2,465.20 *m/z*). Mass tolerance was set to 15 ppm.

### Kinase assays.

In vitro phosphorylation of 1 μg of different purified proteins was carried out in a reaction mixture containing 25 mM Tris-HCl (pH 7.0), 1 mM DTT, 5 mM MgCl_2_, 1 mM EDTA, and 10 μM ATP with 200 μCi/ml [γ-^32^P]ATP. After 10 min incubation at 37 °C, the samples were analyzed by SDS-PAGE electrophoresis. After migration the gels were soaked in 16% TCA for 10 min at 90 °C and stained with Coomassie Blue. The radioactive proteins were visualized by autoradiography using direct film exposure.

### Crystallization.

Crystallization conditions of the CapAB(K55M) mutant protein were determined at 18 °C by screening commercial crystallization kits using a nanodrop crystallization robot (Cartesian). The first crystals were obtained in the Nextal PEG condition 17. The crystals were improved using an additive screen (Hampton Research). The crystal used for diffraction data measurements was obtained at 8.5 mg/ml of CapAB(K55M) in the presence of 10 mM MgCl_2_ after equilibration with 20% (v/v) PEG 1000, 200 mM glycine, and 0.1 M Na-Hepes (pH 7.5). Crystals of CapAB were obtained by manual screening using the hanging drop method. They grew at 35 mg/ml CapAB in presence of 10 mM ATP-Mg after equilibration against a crystallization solution containing 23% (v/v) PEG 1000 and 0.1 M Tris-HCl (pH 8.8).

### Structural determination.

Crystals were frozen in liquid nitrogen, after soaking in a cryoprotectant solution consisting in reservoir solution supplemented with increasing glycerol concentration up to 25% (w/v). Diffraction data were collected at the European Synchrotron Radiation Facility (ESRF) in Grenoble, France, on the microfocus beamline ID23–2 and on beamline ID29 for CapAB and CapAB(K55M), respectively.

The CapAB crystal system is monoclinic, space group P1, with two molecules per asymmetric unit. Data from two crystals were merged and scaled with the XDS package [57]. The structure was solved by molecular replacement with PHASER [58] using the P. horikoshii cell division regulator MinD [38] as the search model. The initial solution of CapAB was then rebuilt with ARP/wARP [59].

The CapAB(K55M) crystal system is I4, with two molecules per asymmetric unit. Data were processed and scaled with the MOSFLM package [60]. The structure was solved by molecular replacement with PHASER [58] using the wild-type CapAB as the search model.

Models were visualised and built using COOT [61]. The refinements were done using CNS [62] and REFMAC [63], and monitored using the free R factor. The final models were evaluated using COOT validation tools and PROCHECK. A summary of the refinement and data statistics is given in Table 1.

## Supporting Information

Figure S1 CapAB / MinD Structure Comparison (A) Structure superimposition. CapAB is represented with CapB coloured in spectrum and CapACt in grey. P. horikoshii MinD is shown in beige. A circular permutation led to the superimposition of the MinD 60-residue–long C-terminal extension (α8-β8-α9-α10-α11) with the N-terminal part of the CapAB chimer, i.e., the 29 residues of CapACt and a 40-residue–long N-terminal extension of CapB. Hence, MinD fragment α8-β8 superimposes with CapACt αA-βA. MinD helix α9 corresponds to the loop linking CapACt to CapB, and the MinD fragment α10–α11 superimposes with α1–α2 of CapB. Finally, all the secondary structure elements are conserved with different topologies.
(B) Structure-based sequence alignment. The circular permutation allowing the accommodation of the C-terminal tyrosine cluster (green box) of CapAB is highlighted by the superimposition of the MinD sequence on two consecutive sequences of CapAB.
(3.5 MB EPS)

Figure S2 Functional Analysis of the CapA Residue F221a The autophosphorylation activity of the CapAB chimeric protein (WT) and of the F221 mutated form (F221A) was analyzed by SDS-PAGE and autoradiography.
(399 KB EPS)

Figure S3 BY-Kinases Sequence Comparison The cytoplasmic region of the associated transmembrane activator has been added to all representatives of the firmicute BY-Kinases in order to mimic the S. aureus CapAB chimera. YwqCD represents Bacillus subtilis YwqC/YwqD, and YveKL B. subtilis YveK/YveL. Only the C-terminal part of proteobacterial BY-kinase sequences are listed: E. coli Wzc and Etk; Acinetobacter johnsonii Ptk, and three BY-kinases (BYK) from Erwinia amylovora, Ralstonia solanacearum, and *Stigmatella aurantiaca*. Important residues are highlighted with signs at the bottom of the alignment: P-loop residues with red square; residues involved in octamer contacts with green stars; residues involved in adenine base binding with blue triangles; residues chelating the magnesium ion with white circles; residues involved in Tyr225 interaction with orange circles; and Tyr225 with a cyan triangle.
(16 KB PDF)

Figure S4 Phosphorylation of CapO Protein CapO was incubated in the presence of radioactive ATP with either the wild-type form of CapAB (WT), the truncated form missing the C-terminal tyrosine cluster (Cut), or the substituted form of the cluster with Glu mimicking the phosphorylated tyrosines (YCE). Phosphorylation was then analyzed by SDS-PAGE and autoradiography
(372 KB EPS)

Table S1 Oligonucleotides Used in This Study (24 KB DOC)

### Accession Numbers

Coordinates and data of the CapAB and CapAB(K55M) structures have been deposited into the Protein Data Bank (PDB; http://www.rcsb.org/pdb) with ID codes 3BFV and 2VED, respectively. The PDB assession number for the MinD protein is 1ION, and for the MinD/AMP-PCP complex is 1G3R.

Accession numbers from the National Center for Biotechnology Information (NCBI; http://www.ncbi.nlm.nih.gov) for proteins mentioned in this paper are Bacillus subtilis YveK (CAB15442), YveL (CAB15441), YwqC (CAB15643), and YwqD (CAB15642).

## Abbreviations

BY-kinase: bacterial tyrosine kinase
EM: electron microscopy
PCP: polysaccharide copolymerase
