# Supplementary material for: Structural Basis for the Regulation Mechanism of the Tyrosine Kinase CapB from Staphylococcus aureus
Source: PLoS Biol. 2008 Jun 10;6(6):e143. doi: 10.1371/journal.pbio.0060143 (PMC2422856; doi:10.1371/journal.pbio.0060143)
Supplement: Supplementary file 1 [file pbio.0060143.sg003.pdf]

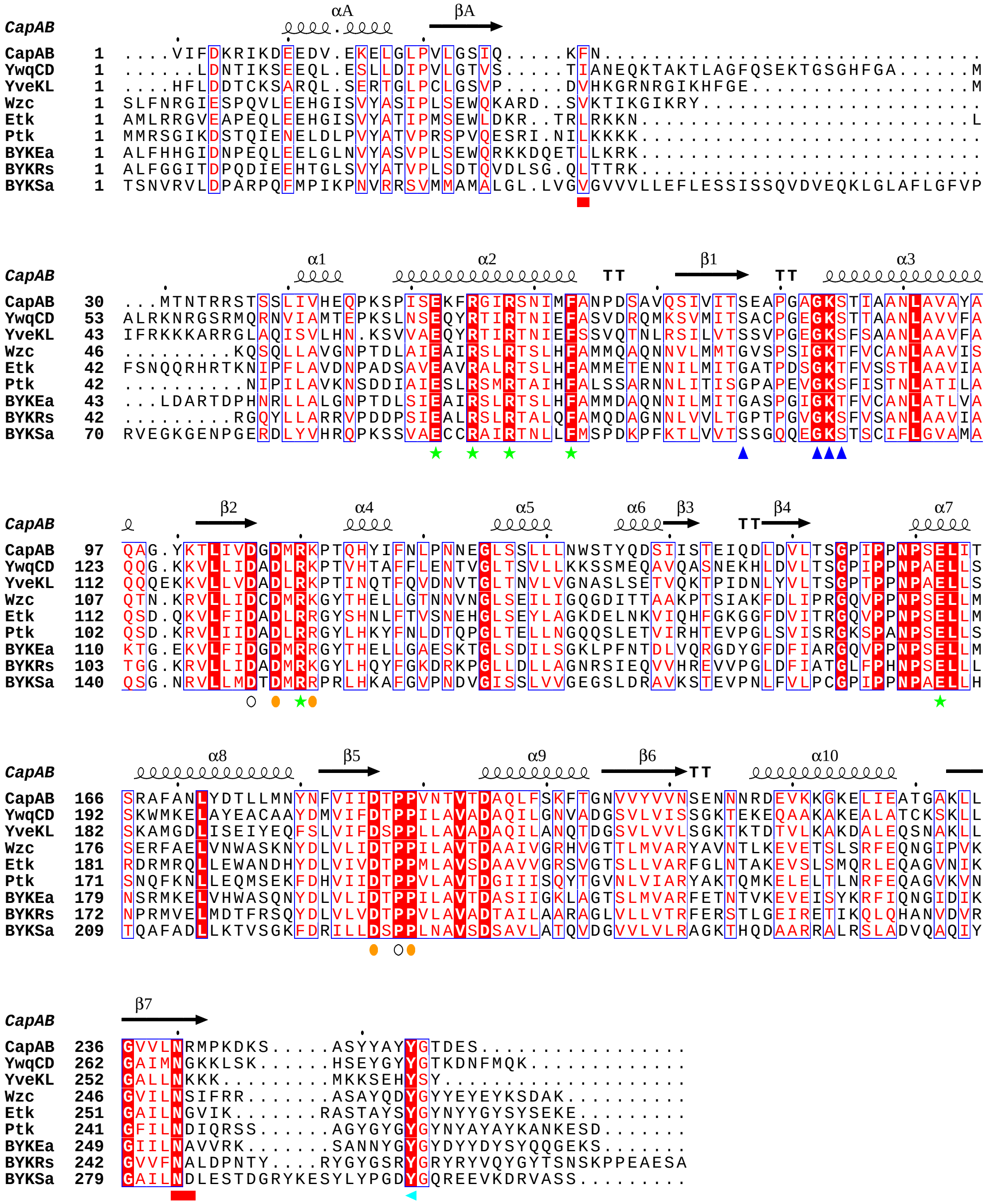

CapAB αA βA
CapAB 1 ....VIFDKRIKDEEDV.EKELGLPVLGSIQ.....KFN...............................
YwqCD 1 ......LDNTIKSEEQL.ESLLDIPVLGTVS.....TIANEQKTAKTLAGFQSEKTGSGHFGA......M
YveKL 1 ....HFLDDTCKSARQL.SERTGLPCLGSVP.....DVHKGRNRGIKHFGE..................M
Wzc 1 SLFNRGIESPQVLEEHGISVYASIPLSEWQKARD..SVKTIKGIKRY.......................
Etk 1 AMLRRGVEAPEQLEEHGISVYATIPMSEWLDKR..TRLRKKN...........................L
Ptk 1 MMRSGIKDSTQIENELDLPVYATVPRSPVQESRI.NILKKKK............................
BYKEa 1 ALFHHGIDNPEQLEELGLNVYASVPLSEWQRKKDQETLLKRK............................
BYKRs 1 ALFGGITDPQDIEEHTGLSVYATVPLSDTQVDLSG.QLTTRK............................
BYKSa 1 TSNVRVLDPARPQFMPIKPNVRRSVMMAMALGL.LVGVGVVVLLEFLESSISSQVDVEQKLGLAFLGFVP
CapAB α1 α2 TT β1 TT α3
CapAB 30 ...MTNTRRSTSSLIVHEQPKSPISEKFRGIRSNIMFANPDSAVQSIVITSEAPGAGKSTIAANLAVAYA
YwqCD 53 ALRKNRGSRMQRNVIAMTEPKSLNSEQYRTIRTNIEFASVDRQMKSVMITSACPGEGKSTTAANLAVVFA
YveKL 43 IFRKKKARRGLAQISVLHN.KSVVAEQYRTIRTNIEFSSVQTNLRSILVTSSVPGEGKSFSAANLAAVFA
Wzc 46 .........KQSQLLAVGNPTDLAIEAIRSLRTSLHFAMMQAQNNVLMMTGVSPSIGKTFVCANLAAVIS
Etk 42 FSNQQRHRTKNIPFLAVDNPADSAVEAVRALRTSLHFAMMETENNILMITGATPDSGKTFVSSTLAAVIA
Ptk 42 ..........NIPILAVKNSDDIAIESLRSMRTAIHFALSSARNNLITISGPAPEVGKSFISTNLATILA
BYKEa 43 ...LDARTDPHNRLLALGNPTDLSIEAIRSLRTSLHFAMMDAQNNILMITGASPGIGKTFVCANLATLVA
BYKRs 42 .........RGQYLLARRVPDDPSIEALRSLRTALQFAMQDAGNNLVVLTGPTPGVGKSFVSANLAAVIA
BYKSa 70 RVEGKGENPGERDLYVHRQPKSSVAECCRAIRTNLLFMSPDKPFKTLVVTSSGQQEGKSTSCIFLGVAMA
CapAB β2 α4 α5 α6 β3 TT β4 α7
CapAB 97 QAG.YKTLIVDGDMRKPTQHYIFNLPNNEGLSSLLLNWSTYQDSIISTEIQDLDVLTSGPIPPNPSELIT
YwqCD 123 QQG.KKVLLIDADLRKPTVHTAFFLENTVGLTSVLLKKSSMEQAVQASNEKHLDVLTSGPIPPNPAELLS
YveKL 112 QQQEKKVLLVDADLRKPTINQTFQVDNVTGLTNVLVGNASLSETVQKTPIDNLYVLTSGPTPPNPAELLS
Wzc 107 QTN.KRVLLIDCDMRKGYTHELLGTNNVNGLSEILIGQGDITTAAKPTSIAKFDLIPRGQVPPNPSELLM
Etk 112 QSD.QKVLFIDADLRRGYSHNLFTVSNEHGLSEYLAGKDELNKVIQHFGKGGFDVITRGQVPPNPSELLM
Ptk 102 QSD.KRVLIIDADLRRGYLHKYFNLDTQPGLTELLNGQQSLETVIRHTEVPGLSVISRGKSPANPSELLS
BYKEa 110 KTG.EKVLFIDGDMRRGYTHELLGAESKTGLSDILSGKLPFNTDLVQRGDYGFDFIARGQVPPNPSELLM
BYKRs 103 TGG.KRVLLIDADMRKGYLHQYFGKDRKPGLLDLLAGNRSIEQVVHREVVPGLDFIATGLFPHNPSELLL
BYKSa 140 QSG.NRVLLMDTDMRRPRLHKAFGVPNDVGISSLVVGEGSLDRAVKSTEVPNLFVLPCGPIPPNPAELLH
CapAB α8 β5 α9 β6 TT α10
CapAB 166 SRAFANLYDTLLMNYNFVIIDTPPVNTVTDAQLFSKFTGNVVYVVNSENNNRDEVKKGKELIEATGAKLL
YwqCD 192 SKWMKELAYEACAAYDMVIFDTPPILAVADAQILGNVADGSVLVISSGKTEKEQAAKAKEALATCKSKLL
YveKL 182 SKAMGDLISEIYEQFSLVIFDSPPLLAVADAQILANQTDGSVLVVLSGKTKTDTVLKAKDALEQSNAKLL
Wzc 176 SERFAELVNWASKNYDLVLIDTPPILAVTDAAIVGRHVGTTLMVARYAVNTLKEVETSLSRFEQNGIPVK
Etk 181 RDRMRQLLEWANDHYDLVIVDTPPMLAVSDAAVVGRSVGTSLLVARFGLNTAKEVSLSMQRLEQAGVNIK
Ptk 171 SNQFKNLLEQMSEKFDHVIIDTPPVLAVTDGIIISQYTGVNLVIARYAKTQMKELELTLNRFEQAGVKVN
BYKEa 179 NSRMKELVHWASQNYDLVLIDTPPILAVTDASIIGKLAGTSLMVARFETNTVKEVEISYKRFIQNGIDIK
BYKRs 172 NPRMVELMDTFRSQYDLVLVDTPPVLAVADTAILAARAGLVLLVTRFERSTLGEIRETIKQLQHANVDVR
BYKSa 209 TQAFADLLKTVSGKFDRILLDSPPLNAVSDSAVLATQVDGVVLVLRAGKTHQDAARRALRSLADVQAQIY
CapAB β7
CapAB 236 GVVLNRMPKDKS.....ASYYAYYGTDES.................
YwqCD 262 GAIMNGKKLSK......HSEYGYYGTKDNFMQK.............
YveKL 252 GALLNKKK.........MKKSEHYSY....................
Wzc 246 GVILNSIFRR.......ASAYQDYGYYEYEYKSDAK..........
Etk 251 GAILNGVIK.......RASTAYSYGYNYYGYSYSEKE.........
Ptk 241 GFILNDIQRSS......AGYGYGYGYNYAYAYKANKESD.......
BYKEa 249 GIILNAVVRK.......SANNYGYGYDYYDYSYQQGEKS.......
BYKRs 242 GVVFNALDPNTY....RYGYGSRYGRYRYVQYGYTSNSKPPEAESA
BYKSa 279 GAILNDLESTDGRYKESYLYPGDYGQREEVKDRVASS.........
