# Supplementary material for: Structural Basis for the Regulation Mechanism of the Tyrosine Kinase CapB from Staphylococcus aureus
Source: PLoS Biol. 2008 Jun 10;6(6):e143. doi: 10.1371/journal.pbio.0060143 (PMC2422856; doi:10.1371/journal.pbio.0060143)
Supplement: Supplementary file 2 [file pbio.0060143.st001.doc]

Supplementary Table 1 . Oligonucleotides used in this  study

Primer^c^	5' to 3' Sequence  ^a,b^	
Y221E(-)	TATAAGCTTTCATGATTCATCAGTCCCATAATATGCATATTCACTAGCACTTTTATCTTTAGGC	
Y221F(-)	TATAAGCTTTCATGATTCATCAGTCCCATAATATGCATAAAAACTAGCACTTTTATCTTTAGGC	
Y225E(-)	TATAAGCTTTCATGATTCATCAGTCCCTTCATATGCATAGTAAC	
Y225F(-)	TATAAGCTTTCATGATTCATCAGTCCCAAAATATGCATAGTAAC	
Y221-222E(-)	TATAAGCTTTCATGATTCATCAGTCCCATAATATGCTTCTTCACTAGCACTTTTATCTTTAGGC	
Y221-222F(-)	TATAAGCTTTCATGATTCATCAGTCCCATAATATGCAAAAAAACTAGCACTTTTATCTTTAGGC	
Y224-225E(-)	TATAAGCTTTCATGATTCATCAGTCCCTTCTTCTGCATAGTAACTAGCACTTTTATCTTTAGGC	
Y224-225F(-)	TATAAGCTTTCATGATTCATCAGTCCCAAAAAATGCATAGTAACTAGCACTTTTATCTTTAGGC	
Y221-222-224E(-)	TATAAGCTTTCATGATTCATCAGTCCCATATTCTGCTTCTTCACTAGCACTTTTATCTTTAGGC	
Y221-222-224F(-)	TATAAGCTTTCATGATTCATCAGTCCCATAAAATGCAAAAAAACTAGCACTTTTATCTTTAGGC	
Y225-224-222E(-)	TATAAGCTTTCATGATTCATCAGTCCCTTCTTCTGCTTCGTAACTAGCACTTTTATCTTTAGGC	
Y225-224-222F(-)	TATAAGCTTTCATGATTCATCAGTCCCAAAAAATGCAAAGTAACTAGCACTTTTATCTTTAGGC	
Y225-224-222-221E	TATAAGCTTTCATGATTCATCAGTCCCTTCTTCTGCTTCTTCACTAGCACTTTTATCTTTAGGC	
Y225-224-222-221F	TATAAGCTTTCATGATTCATCAGTCCCAAAAAATGCAAAGAAACTAGCACTTTTATCTTTAGGC	
E23A-R26A-R29A(-)	CATAATATTTGATGCTATGCCTGCAAATTTCGCGCTAATAGGTGACTTTGG	
F221A(-)	GTATTCGTCATATTAGCTTTTTGAATTGAACCC	
AB-Cut(-)	TATAAGCTTTTAACTAGCACTTTTATCTTTAGGC	
Nt-CapAB(+)	TATGGATCCAAAGTAATTTTCGATAAGCGAATT	
Nt-CapC(+)	GGAATTCCATATGATTGATATTCATAACCATATA	
Ct-CapC(-)	CGGGATCCTTAAAACCATTTCAAAAACGA	
^a^ mutated bases are bolded.
^b^ Restriction sites are italicized.
^c^ Forward and reverse primers are represented by plus (+) or minus (-),respectively.
